# A54 INFLAMMATORY BOWEL DISEASE IN INDIGENOUS POPULATIONS: A SCOPING REVIEW

**DOI:** 10.1093/jcag/gwae059.054

**Published:** 2025-02-10

**Authors:** O Munir, C Condray, A P Souza Lira, S Fowler, C Brass, H Butterworth, A Hourston, L Porter, R Porter, R Sanderson, J Peña-Sánchez

**Affiliations:** University of Saskatchewan College of Medicine, Saskatoon, SK, Canada; The University of Oklahoma, Norman, OK; University of Saskatchewan College of Medicine, Saskatoon, SK, Canada; University of Saskatchewan College of Medicine, Saskatoon, SK, Canada; Muskoday First Nation, Muskoday First Nation, SK, Canada; White Fish River First Nation, Whitefish River First Nation, ON, Canada; Wasauksing First Nation, Wasauksing First Nation, ON, Canada; One Arrow First Nation, One Arrow First Nation, SK, Canada; York Factory First Nation, York Factory First Nation, MB, Canada; James Smith Cree Nation, James Smith Cree Nation, SK, Canada; University of Saskatchewan College of Medicine, Saskatoon, SK, Canada

## Abstract

**Background:**

Globally, Inflammatory Bowel Disease (IBD) rates have surged; however, Indigenous/Native/Aboriginal populations (i.e., the original inhabitants of a geographical region) are underrepresented in research and often face unique challenges in accessing healthcare due to socioeconomic barriers stemming from historic inequities.

**Aims:**

This scoping review aimed to synthesize available literature on IBD in Indigenous populations globally, identify research gaps, and propose recommendations to improve research inclusivity.

**Methods:**

A literature search was conducted across eight online databases: MEDLINE, EMBASE, CINAHL, SCOPUS, Web of Science, i-portal, Native Health Database, and ASTIS. We included qualitative, quantitative, and mixed-method research in this review, as well as commentaries, editorials, and abstracts published between 1960 and 2023. Eligibility criteria included publications in English focused on IBD Indigenous populations. Two reviewers independently screened titles and abstracts. The included publications were summarized and appraised using the Joanna Briggs Institute critical appraisal tools.

**Results:**

Eighteen publications were included in the review, with most originating from Canada (n=7), New Zealand (n=5), and Australia (n=4). There was one study from Chile and another from the United States. Results showed a lower prevalence of IBD in Indigenous populations compared to the general population, but some recent studies reported an increasing prevalence of IBD among Indigenous populations from different countries. These changes are hypothesized to result from urbanization, changes in diet, sanitation, or other environmental changes. Also, a study from Canada showed that Indigenous peoples face inequities in accessing IBD care. Apart from 3 papers from Canada, 15 of the 18 publications had no evidence of working with Indigenous community members during the research process.

**Conclusions:**

This scoping review highlights gaps in the literature about IBD in Indigenous populations. While the prevalence of IBD among Indigenous peoples is low, evidence demonstrates that rates are rising. Further research is required to continue studying the rising rates of IBD in Indigenous populations, alongside the genetic and environmental factors contributing to these trends. A focus on patient-centered research should also be emphasized. Indigenous peoples must be included as research partners and Indigenous research protocols must be followed to address health inequities and improve the health and well-being of Indigenous peoples living with IBD.

**Funding Agencies:**

None

